# ^64^Cu-NODAGA-c(RGDyK) Is a Promising New Angiogenesis PET Tracer: Correlation between Tumor Uptake and Integrin **α**_*V*_ **β**_3_ Expression in Human Neuroendocrine Tumor Xenografts

**DOI:** 10.1155/2012/379807

**Published:** 2012-10-02

**Authors:** Jytte Oxboel, Christina Schjoeth-Eskesen, Henrik H. El-Ali, Jacob Madsen, Andreas Kjaer

**Affiliations:** Department of Clinical Physiology, Nuclear Medicine & PET, Rigshospitalet and Cluster for Molecular Imaging, Faculty of Health Sciences, University of Copenhagen, Copenhagen, Denmark

## Abstract

*Purpose*. The purpose of this paper is to evaluate a new PET tracer ^64^Cu-NODAGA-c(RGDyK) for imaging of tumor angiogenesis using gene expression of angiogenesis markers as reference and to estimate radiation dosimetry for humans. *Procedures*. Nude mice with human neuroendocrine tumor xenografts (H727) were administered ^64^Cu-NODAGA-c(RGDyK) i.v. for study of biodistribution as well as for dynamic PET. Gene expression of angiogenesis markers integrin *α*_*V*_, integrin *β*_3_, and VEGF-A were analyzed using QPCR and correlated to the tracer uptake in the tumors (%ID/g). From biodistribution data human radiation-absorbed doses were estimated using OLINDA/EXM. *Results*. Tumor uptake was 1.2%ID/g with strong correlations between gene expression and tracer uptake, for integrin *α*_*V*_ *R* = 0.76, integrin *β*_3_ *R* = 0.75 and VEGF-A *R* = 0.81 (all *P* < 0.05). The whole body effective dose for humans was estimated to be 0.038 and 0.029 mSv/MBq for females and males, respectively, with highest absorbed dose in bladder wall. *Conclusion*. ^64^Cu-NODAGA-c(RGDyK) is a promising new angiogenesis PET tracer with potential for human use.

## 1. Introduction

Angiogenesis, the formation of new blood vessels, plays an important role in tumor growth, local invasiveness, and metastatic progression. Many preclinical studies and clinical trials confirm the importance of integrin *α*_*V*_ *β*_3_ in the process of tumor angiogenesis and metastasis [1, 2]. Integrin *α*_*V*_ *β*_3_ is a cell adhesion molecule and is highly expressed on activated endothelial cells and tumor cells but is not expressed on resting endothelial cells and therefore specific for neoangiogenesis [3]. Several extracellular matrix (ECM) proteins like vitronectin, fibrinogen, and fibronectin interact with integrin *α*_*V*_ *β*_3_ via the amino acid sequence Arg-Gly-Asp (RGD) [4, 5]. Vascular endothelial growth factor (VEGF-A) also plays an important role in both normal vascular tissue development and tumor neovascularization and is highly expressed in various human tumors [6, 7]. 

In the last few years metal PET isotopes such as ^68^Ga and ^64^Cu have gained increased interest for labeling of peptides. Both ^68^Ga and ^64^Cu are excellent alternatives to ^18^F. ^68^Ga can be obtained from an in-house commercially available ^68^Ge/^68^Ga generator. The advantage of using cyclotron produced ^64^Cu for PET imaging and radiotherapy is the longer half-life allowing imaging at late time points acquiring additional information [8]. A longer half-life also allows more nonspecific activity to be washed out. ^64^Cu can now be produced in high yield and at high specific activity on a small biomedical cyclotron. 

Radiolabeled RGD peptides for positron emission tomography (PET) imaging, targeting integrin *α*_*V*_ *β*_3_ in tumors, have been described in several studies with mainly ^18^F (*t*_1/2_ = 110 min; *E*_max⁡, *β*+_ = 634 (97%) keV), ^68^Ga (*t*_1/2_ = 68 min; *E*_max⁡, *β*+_ = 1.90 (89%) keV), and ^64^Cu (*t*_1/2_ = 12.7 h; *E*_max⁡, *β*+_ = 653 (18%) keV) [9–17]. For ^68^Ga and ^64^Cu different chelating systems have been used. Some of the latest described tracers have used the chelator 1,4,7-triazacyclononane-1-glutaric acid-4,7-diacetic acid (NODAGA) combined with c(RGD) labeled with ^68^Ga [9, 12, 15, 18] or ^64^Cu [12, 18].

Neuroendocrine tumors (NET) express integrin *α*_*V*_ *β*_3_ at variable levels [19]. NET is one of the tumor types where antiangiogenesis treatment of patients seems promising [20] but patients need to be selected individually. To select the patients, PET scans with a tracer targeting tumor-angiogenesis may be extremely relevant. Therefore using a human NET xenograft model for preclinical studies to evaluate new PET tracers targeting tumor-angiogenesis is relevant.

The aim of the present study was therefore, as the first, to use and evaluate the PET tracer ^64^Cu-NODAGA-c(RGDyK) using a human NET xenograft model. Biodistribution, PET/CT scans, correlation between gene expression of angiogenesis markers and tracer uptake, and radiation dosimetry extrapolation for humans were performed. Since investigation of tumor-angiogenesis using human xenografts in mice is challenged by the fact that the formation of new vessels in the tumors might be of either human or mice (host) origin, we designed QPCR assays to quantify both mice and human genes of Integrin *α*_*V*_, Integrin *β*_3_, and VEGF-A.

## 2. Materials and Methods

### 2.1. ^64^Cu-NODAGA-c(RGDyK) Synthesis

#### 2.1.1. General

NODAGA-c(RGDyK) was purchased from ABX GmbH (advanced biochemical compounds, Radeberg, Germany). Ammonium acetate and TraceSELECT water were purchased from Sigma-Aldrich. ^64^CuCl_2_ was produced at Risoe Technical University of Denmark, Roskilde, Denmark. For high-performance liquid chromatography (HPLC) analysis a Dionex P580 pump with a PDA-100 detector and an in-line Scansys radioactivity detector was used.

#### 2.1.2. Radiochemistry

**Figure 1:**
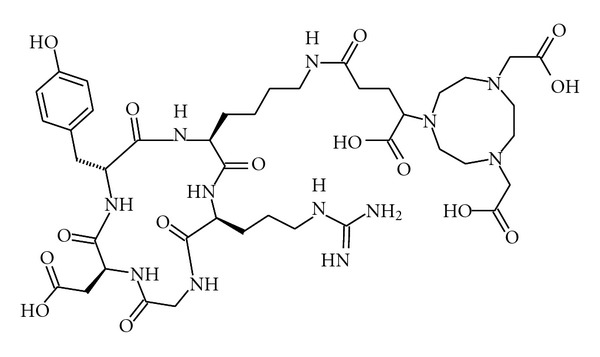
The chemical structure of NODAGA-c(RGDyK).

NODAGA-c(RGDyK) (2 nmol) dissolved in 450 *μ*L ammonium acetate (0.1 M, pH 8.47) was mixed with 50 *μ*L of ^64^CuCl_2_ (50–60 MBq) for 15 min at room temperature. The product (Figure 1) was used without further purification. HPLC analysis was performed with a Jupiter column, 4u Proteo 90A, 250 × 4.6 mm (Phenomenex, Torrance, CA, USA) and flow 1.5 mL/min. The HPLC mobile phase was solvent A, 0.1% trifluoroacetic acid in water, and solvent B, 0.1% trifluoroacetic acid in acetonitrile. Gradient: 0–2 min 15% B, 2–7 min 15–40% B, and 7–10 min 40–15% B. The retention time of ^64^Cu-NODAGA-c(RGDyK) was 5.9 min. 

#### 2.1.3. Stability Studies

The stability of ^64^Cu-NODAGA-c(RGDyK) was investigated in either buffer or plasma from nude mice. For buffer stability, 160 MBq ^64^Cu-NODAGA-c(RGDyK) was incubated for 1, 2, 18, and 24 hours at room temperature in 450 *μ*L ammonium acetate buffer. For the stability in plasma, 10 MBq ^64^Cu-NODAGA-c(RGDyK) was incubated for 1, 2, 18, and 24 hours in 500 *μ*L plasma. Plasma was mixed with equal amounts of acetonitrile after incubation. The resulting mixture was centrifugated and the supernatant was collected and filtered. The stability of ^64^Cu-NODAGA-c(RGDyK) in plasma and buffer was analyzed and determined by the previously described HPLC conditions.

### 2.2. Cell Line and Animal Model

Human lung bronchus carcinoid, NCI-H727 obtained from ATCC (American Type Culture Collection, Manassas, VA, USA), was used. Before taken into experiments, the cell line was tested free of Mycoplasma at Statens Serum Institute, Copenhagen, Denmark. Cells were cultured in RPMI (Roswell Park Memorial Institute) Medium 1640 with GlutaMAX (Gibco, Life Technologies, NY, USA) containing 10% fetal calf serum (Biological Industries (BI), Kibbutz Beit-Haemek, Israel), and 1% penicillin-streptomycin (Gibco, Life Technologies) in 5% CO_2_ at 37°C.

Female NMRI (Naval Medical Research Institute) nude mice (6 weeks upon arrival) were acquired from Taconic Europe, Lille Skensved, Denmark. The mice were acclimated one week in the animal facilities before taken into experiments. At the age of 8 weeks (weight: 32.8 ± 4.2 g) the mice had H727 cells (5–7 × 10^6^ cells in 100 *μ*L medium mixed with 100 *μ*L Matrigel Basement Membrane Matrix (BD Sciences, San José, CA, USA) inoculated subcutaneously on the left and right flank during anesthesia with 1 : 1 V/V Hypnorm^R^ (Janssen Pharmaceutica NV, Beerse, Belgium)/Dormicum^R^  (Roche, Basel, Switzerland). Tumors were then grown for 3-4 weeks (average tumor volume 210 mm^3^). Animal care and experimental procedures were performed under the approval of the Danish Animal Welfare Council (2006/561-1124).

### 2.3. Biodistribution Studies

Tumor bearing mice were anesthetized with 3% sevoflurane (Abbott Scandinavia AB, Solna, Sweden) mixed with 35% O_2_ in N_2_. Mice were tail-vein injected with 2.0 ± 0.7 MBq ^64^Cu-NODAGA-c(RGDyK). Biodistribution of ^64^Cu-NODAGA-c(RGDyK) was obtained at 1, 2, and 18 hours after injection (*n* = 5 mice in each group). Mice were sacrificed by neck drawing and tumors, organs (liver, kidneys, lung, spleen, heart, intestine, and muscle) and blood were collected, weighted, and radioactivity measured in a gamma counter (2480 Wizard^2^, Perkin Elmer, MA, USA). Tumors were immediately placed in tubes containing RNAlater^R^ (Ambion Inc., TX, USA), stored overnight at 4°C, RNAlater^R^ then removed, and tumors stored at −80°C until total RNA extraction and further QPCR experiments were performed.

### 2.4. Dosimetry Extrapolation for Humans

For estimation of the human radiation dose using ^64^Cu-NODAGA-c(RGDyK) the mouse biodistribution data were used. Assuming similar pharmacokinetics in mice and humans, the activity in organs at 1, 2, and 18 hours post injection (p.i.) was used to calculate single exponential effective half-lives and residence times for each organ. These data were then used for extrapolation to human dosimetry estimates using standard female (58 kg) and male (70 kg) human phantoms and the Organ Level INternal Dose Assessment/EXponential Modeling software (OLINDA/EXM; Vanderbilt University, Nashville, TN, USA) [21]. To ensure a conservative estimate, the urinary elimination fraction was set to 75% with voiding intervals of 5 hours. The effective dose for both adult male and female were then calculated using OLINDA/EXM.

### 2.5. Small Animal PET and CT 

#### 2.5.1. Imaging Experiments

A longitudinal dynamic study was performed scanning a mouse at 1, 2, and 18 hours after intravenous injection of 1.6 MBq ^64^Cu-NODAGA-c(RGDyK), allowing dynamic information of the tracer biodistribution in tumors as well as in the other organs of interest. The PET scans were performed using a small animal PET scanner (MicroPET Focus 120, Siemens Medical Solutions, Knoxville, TN, USA). The energy window for the emission PET scans was set to 350–650 keV and the time resolution was 6 ns. Scan time was 10 min.

The acquired data for emission scan was stored in list-mode format and postprocessed to obtain 2 bytes 128 × 144 × 32 sinograms. Finally, the emission sinograms were reconstructed using MAP Algorithms and resulted into 4-byte 256 × 256 × 95 image sets with a zoom factor of 1.443 and a voxel size of 0.87 × 0.87 × 0.79 mm^3^. Furthermore, the emission sinograms were corrected for dead time and decay time. Scatter and attenuation corrections were not applied to the emission data. The system was calibrated to provide activity concentrations as Bq/cc. 

The small animal CT scans of the mouse parallel with the PET scans were acquired using a small animal computed tomography (microCAT II, Siemens Medical Solutions). The acquisition time of each CT scan was 6.5 minutes generating 360 projections at 360° arc. The X-ray source settings were 75 kVp, 500 *μ*A, and 270 ms for the voltage, the current, and the exposure time, respectively. The CT projections on the 3,000 × 2,970 CCD crystal were binned by 4 to increase sensitivity and reduce the dataset size. The projections were reconstructed by real-time reconstruction algorithm (the COBRA toolbox) using Shepp-Logan filter into 768 × 768 × 512 matrix with a voxel size of 0.092 × 0.092 × 0.092 mm^3^. 

#### 2.5.2. Image Analysis

The fusion of the PET and CT images was performed manually using the ASIPro Toolbox (Siemens Medical Solutions). Also the analyses of PET images were performed in the ASIPro toolbox. 

The measurement of the activity uptake in the tumors and the organs were estimated by manually outlining on CT images. The outlining of the organs on CT images in every slice resulted in 3D region of interest (ROI). The 3D ROIs of the organs were then applied on the corresponding PET images. The results of these ROIs acquired the concentration of the tracer in the organs expressed in percentage of the injected dose per gram tissue (%ID/g).

### 2.6. Quantitative Real-Time QPCR

#### 2.6.1. RNA Extraction and Reverse Transcription

The whole tumors were lysed and homogenized in Precellys^R^-24 (Bertin Technologies, Montigny, France) in tubes containing ceramic beads. Total RNA was isolated using NucleoSpin RNA L kit (MACHEREY-NAGEL GmbH & Co. KG, Düren, Germany). RNA concentration was determined by NanoDrop 1000 (Thermo Fisher Scientific, DE, USA). RNA quality expressed as RNA Integrity Number (RIN) was measured on a 2100 Bioanalyzer (Agilent Technologies, CA, USA). 0.3 *μ*g total RNA was reverse transcribed using AffinityScript QPCR cDNA Synthesis Kit (Stratagene, CA, USA). 

#### 2.6.2. Quantitative Real-Time PCR

Gene expression was quantified on the Mx3005P real-time PCR system from Stratagene. Human ITGAV, human ITGB3 and human VEGFA were tested in a triplex-, mice ITGAV and mice ITGB3 in a duplex, and mice VEGFA in a simplex-QPCR assay. The three human housekeeping genes HPRT, UBC, and RPLP were also measured in a triplex. The Brilliant QPCR Core Reagent Kit (Stratagene; cat# 600530) was used for all the multiplex assays. Assay optimization resulted in a 50% and 100% increase of Taq polymerase and dNTP in duplex and triplex assays, respectively. An MgCl_2_ concentration of 5.5 mM was optimal in all designs. The Brilliant III Ultra-Fast QPCR Master Mix (Stratagene, Cat no. 600880) was used for simplex assay. NO-RT (no reverse transcription) for all the samples was tested using the housekeeping gene-triplex. The thermal profile for QPCR Core Reagent was: denaturation at 95°C for 10 minutes, followed by 40 cycles with denaturation at 95°C for 30 seconds and annealing/elongation at 60°C for 1 minute. The thermal profile for Brilliant III Reagent was: denaturation at 95°C for 3 minutes, followed by 40 cycles with denaturation at 95°C for 20 seconds and annealing/elongation at 60°C for 20 seconds. 

The samples were run in triplicates using 1 *μ*L of cDNA in a total volume of 25 and 20 *μ*L, respectively.

The optimal housekeeping genes for the H727 tumors were found by testing 12 human reference genes from TATAA Biocenter (Goteborg, SE) in 12 different H727 tumors from NMRI nude mice. With geNorm software [22] the genes and the number of genes giving the most stable endogenous normalization were found. The most stable housekeeping genes were HPRT, UBC, and RPLP. Normalization to these three housekeeping genes was executed for all assays.

#### 2.6.3. Primers and TaqMan Dual-Labeled Probes

The six genes of interest m-ITGAV (NM_008402), m-ITB3 (NM_016780), m-VEGFA (NM_001025250), h-ITGAV (NM_002210), h-ITGB3 (NM_000212), and h-VEGFA (NM_001025366), along with the housekeeping genes h-HPRT (NM_000194), h-UBC (NM_021009) and h-RPLP (NM_001002) were designed using Beacon Designer (Premier BioSoft, CA, USA). Before designing, all genes were tested for cross homology against the human or the mouse genome and checked for secondary structures. For the optimized conditions of all primers and probes see Table 1. All primers and probes were purchased from Sigma-Aldrich (St. Louis, MO, USA). Final QPCR designs are listed in Table 2.

QBasePlus software [23] based on the comparative method (2^−ΔΔC*t*^) [24] for relative quantification was used including normalization to three housekeeping genes. Furthermore an inter-run calibrator for correction between PCR runs within the same gene and an arbitrary overall calibrator for comparison of the gene levels in between mice and human genes were taking into account.

### 2.7. Statistics

As shown in earlier studies [19] the PCR data were found not to be normal distributed when tested by a One-sample Kolmogorov-Smirnov test. All QPCR data were therefore log_10_ transformed to obtain normal distribution and the log-transformed data were used for all the subsequent statistical analyses. 

T-Test (two tailed) was used for comparison of mRNA levels of each gene—human versus mice—at the three different sample collecting times. Correlation between genes and %ID/g were evaluated using linear regression.

Data are presented as mean ± SEM. All statistical analyses were performed using SPSS 17.0 statistical software (SPSS Inc., Chicago, Illinois, USA). *P* < 0.05 was considered significant.

## 3. Results

### 3.1. Radiochemistry

NODAGA-c(RGDyK) was labelled with ^64^Cu in 15 minutes at room temperature with a radiochemical purity of 92.5–96.6% and a specific activity of 25.1–25.7 MBq/nmol. The purity of ^64^Cu-NODAGA-c(RGDyK) in buffer was more than 93% at 1, 2, 18, and 24 hours after incubation. The purity was also more than 93% after 24 hours when 4.5 mL of saline was added to 500 *μ*L of ^64^Cu-NODAGA-c(RGDyK) solution. In plasma, the purity of ^64^Cu-NODAGA-c(RGDyK) was 88% after 24 hours of incubation.

### 3.2. Gene Expression of Integrins and VEGFA versus PET Tracer Uptake

#### 3.2.1. RNA

Total RNA concentration in tumors was 0.3 to 1.0 *μ*g/*μ*L. RIN (RNA integrity numbers) were measured to be between 6.8 and 9.9, verifying fine RNA quality with limited degradation. NO-RT for each sample was checked in QPCR, finding no Ct values, indicating that genomic DNA is not present and therefore not disturbing the gene expression values.

#### 3.2.2. QPCR Data

**Figure 2:**
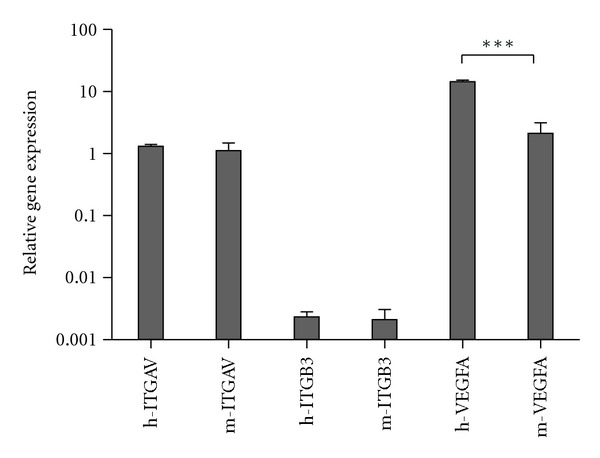
Relative gene expression of human (h) and mouse (m) Integrin *α*_V_ (ITGAV), Integrin *β*_3 _(ITGB3), and VEGFA in H727 tumors in nude mice 2 hours after injection of ^64^Cu-NODAGA-c(RGDyK). Gene expression level for h-VEGFA is significantly higher than the gene expression level for m-VEGFA (*P* < 0.001). No significant difference in gene level was found for the integrins. Please note that the *y*-axis of the graph is in logarithmic scale.

In H727 tumors (removed 2 hours after tracer injection) integrin *α*_V_ of both human and mice origin was expressed at the same level, whereas gene expression of h-VEGF-A was significantly higher than m-VEGF-A (*P* < 0.001). Integrin *β*_3_ of both human and mice origin was expressed on a very low level (Figure 2). 

**Figure 3:**
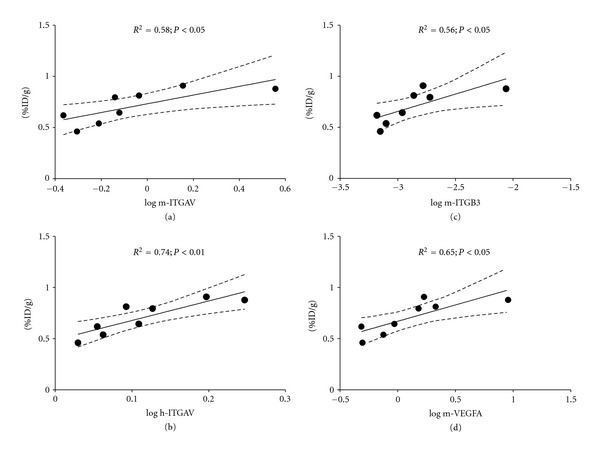
Univariate regression of mouse (m) or human (h) gene expression relative to %ID/g. (a) m-ITGAV, (b) h-ITGAV, (c) m-ITGB3 and (d) m-VEGFA, all versus %ID/g at 2 h after injection of ^64^Cu-NODAGA-c(RGDyK). All relative gene expression results plotted are log⁡_10_ transformed. The 95% confidence interval is indicated by the dotted lines.

Regression analyses for ^64^Cu-NODAGA-c(RGDyK) tracer uptake, %ID/g (calculated from the biodistribution data) versus quantitative mRNA in the H727 tumors showed significant correlation at 2 h p.i. with mice Integrin *α*_*V*_ (*R* = 0.76, *P* < 0.05), mice Integrin *β*_3_ (*R* = 0.75, *P* < 0.05), and mice VEGF-A (*R* = 0.81, *P* < 0.05), also for human Integrin *α*_V_ at 2 h p.i. (*R* = 0.86, *P* < 0.01) (Figure 3).

#### 3.2.3. Biodistribution Data

Liver, kidneys, lung, spleen, heart, intestine, muscle, and blood were collected from 10 mice at the following time points: 1, 2, and 18 hours post injection. All tissues were *γ*-counted 120 sec. and %ID/g was calculated from the measured counts by taking counting efficiency of the *γ*-counter (*F* = 0.0943), half-life time for ^64^Cu, decay from injection to counting time, and tissue weight into account. We found the highest %ID/g (mean ± SEM) at 1 h p.i. for the kidneys, intestines, liver and spleen (2.2 ± 0.11, 1.60 ± 0.16, 1.05 ± 0.17, and 0.94 ± 0.11) decreasing already at 2 h p.i. (0.97 ± 0.03, 0.64 ± 0.04, 0.65 ± 0.04, and 0.62 ± 0.04) and decreasing further at 18 h p.i. (0.51 ± 0.02, 0.43 ± 0.04, 0.43 ± 0.02, and 0.33 ± 0.02). 

**Figure 4:**
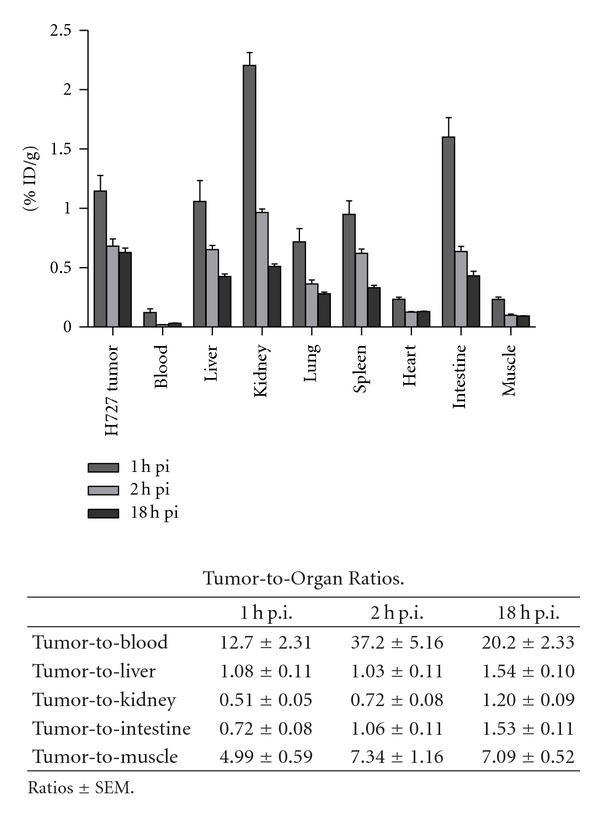
Biodistribution data for ^64^Cu-NODAGA-c(RGDyK) in human H727 xenograft tumor mice at 1, 2 and 18 hour post injection. Results are shown as %ID/g ± SEM.

Mean %ID/g for H727 tumors at 1 h p.i. were 1.15 ± 0.13, at 2 h p.i. 0.68 ± 0.06, and at 18 h p.i. 0.63 ± 0.04. Tumor-to-muscle ratio was calculated to be 6.5 ± 0.7. Tumor-to-blood ratio was 12.7, 37.2, and 20.2, respectively at the 3 collecting times. Biodistribution data and tumor-to-organ ratios are shown in Figure 4.

#### 3.2.4. Radiation Dosimetry

Residence times based on biodistribution data are shown in Table 3 and were used for calculating estimates of human radiation-absorbed doses using OLINDA/EXM.  Table 4 shows the OLINDA/EXM estimates for human adult females and males for each organ and the total effective dose. The highest radiation-absorbed doses were found to be the urinary bladder wall (0.014 and 0.010 mGy/MBq for females and males, resp.). However, this estimate is a worst-case scenario since we used a conservative model with a urinary elimination fraction of 75% with voiding intervals of 5 hours. The whole body effective dose was 0.038 and 0.029 mSv/MBq for females and males, respectively. Accordingly, an administered dose of 200 MBq ^64^Cu-NODAGA-c(RGDyK) to humans would lead to a radiation burden of less than 8 mSv.

#### 3.2.5. PET Scanning Data

**Figure 5:**
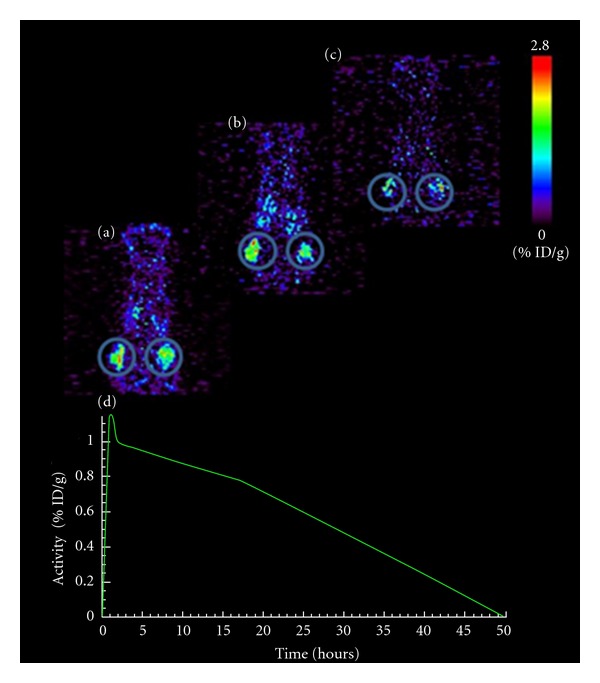
(a)–(c) PET scans showing ^64^Cu-NODAGA-c(RGDyK) uptake in human H727 neuroendocrine tumors in nude mice bearing tumors subcutaneously on the flanks. (a) 1 h post injection of 1.6 MBq ^64^Cu-NODAGA-c(RGDyK) (b) 2 h post injection and (c) 18 h post injection. (d) Time activity curve for ^64^Cu-NODAGA-c(RGDyK) in the H727 tumors calculated as a mean of tracer uptake in left and right tumor at each scan time. Images are 2.5 mm thick slices at the tumor levels; tumors are shown encircled. Scan time: 10 minutes.

The tumors were clearly visible after injection of ^64^Cu-NODAGA-c(RGDyK) at all measured time points: 1, 2 and 18 h pi (Figure 5). ^64^Cu-NODAGA-c(RGDyK) showed relatively high uptake in H727 tumors; mean %ID/g of right and left tumors were 1.15, 0.99, and 0.76 respectively, at 1, 2, and 18 h pi. Tumor-to-muscle (T/M) ratios were 6.9 ± 0.8. Both %ID/g and T/M ratios were similar to the values found in the biodistribution study using the *γ*-counter. Tracer activity in organs did not disturb the tumor to background contrast from 2 h pi where most of the nonspecific uptake had been cleared. 

## 4. Discussion 

To the best of our knowledge this is the first study of the PET tracer ^64^Cu-NODAGA-c(RGDyK). The major finding was a close correlation between tumor uptake of ^64^Cu-NODAGA-c(RGDyK) and the target Integrin *α*_*V*_ *β*_3_ validating its ability to image neo-angiogenesis.

The cyclic RGD peptides c(RGDfK) and c(RDGyK) have frequently been used for radiolabeling. c(RGDfK), cyclo [Arg-Gly-Asp-D-Phe-Lys] only differs from c(RGDyK), cyclo [Arg-Gly-Asp-D-Tyr-Lys], in one amino acid, D-Phenylalanine (D-Phe) instead of D-tyrosine (D-Tyr). D-Tyr is more hydrophilic than D-Phe and c(RGDyK) has therefore been reported as preferable due to faster renal excretion [10]. In our study we found a faster decrease of radioactivity in kidneys, intestine, and liver from 1 to 18 h (2.2 to 0.5, 1.6 to 0.4, and 1.1 to 0.4%ID/g) than that reported for ^64^Cu-NODAGA-c(RGDfK) in the same time intervals (2.2 to 1.6, 2.1 to 1.3, and 1.7 to 1.0%ID/g) [12]. Taking together c(RGDyK) therefore seems superior to c(RGDfK). 

c(RGDyK), the peptide used in the present investigation, has previously been chelated to 1,4,7,10-tetraazacyclododecane-N,N′,N′′,N′′′-tetraacetic acid (DOTA) or 1,4,7-triazacyclononane-1,4,7-triacetic acid (NOTA) and labeled with ^64^Cu [11–13, 25] or ^68^Ga [14]. ^64^Cu-DOTA-c(RGDyK) was first described in 2004 by Chen and coworkers [25]. The ^64^Cu-DOTA complex seemed more unstable than ^64^Cu-NOTA or ^64^Cu-NODAGA, because Cu^2+^ was transferred to copper-binding proteins, leading to relatively high blood and liver uptake [26]. NODAGA is, compared to the chelators DOTA and NOTA [8, 11, 27–29], particularly useful due to high hydrophilicity leading to rapid renal excretion avoiding radioactivity accumulation in the gastrointestinal tract [30] and in the kidneys. Therefore, we chose NODAGA as chelator.

NODAGA-c(RGDy/fK) has been labeled with ^68^Ga [9, 12, 15, 18] and NODAGA-c(RGDfK) also with ^64^Cu [12]. One paper compared ^68^Ga- and ^64^Cu-NODAGA-c(RGDfK) and found the highest tracer uptake using ^68^Ga-NODAGA-c(RGDfK) compared to ^64^Cu-NODAGA-c(RGDfK) (5.2 versus 3.8%ID/g, 1 h p.i. in highly angiogenic U87MG xenograft tumors) but also 3 times higher tumor-to-blood ratio [12]. We found similar T/B ratio (12.7) after 1 hour using ^64^Cu-NODAGA-c(RGDyK) as they did with ^64^Cu-NODAGA-c(RGDfK). The advantage of ^64^Cu labeling is the possibility of delayed imaging up to 12 or 18 h post injection with expected improved image contrast, and therefore improved visualization of low expressing Integrin *α*_V_ *β*_3_ tumors. Indeed, we found that delayed images after 2 and 18 hours showed higher tumor-to-background ratios compared to 1 hour images. This confirms the value of delayed images, possible with ^64^Cu. In addition ^64^Cu has a much better image quality than ^68^Ga due to the shorter positron range. A comparison to ^18^F-labeled RGD shows that tumor-to-liver and tumor-to-kidney ratios are similar [11].

For the neuroendocrine H727 xenograft tumors we found moderate levels of tracer uptake (1.2%ID/g 1 h p.i. as the highest) explained by the fact that neo angiogenic activity in H727 tumors, reflecting low aggressiveness in general of NET tumors, is known to be lower than, for example, in glioblastoma U87MG xenograft tumors showing high expression of integrins [15]. 

We investigated the target specificity of the PET tracer by analyzing mRNA levels of both Integrin *α*_V_ and Integrin *β*_3_ using quantitative QPCR. Our results strongly support that ^64^Cu-NODAGA-c(RGDyK) has high affinity to Integrin *α*_V_ *β*_3_ reflecting neo angiogenesis.

A challenge in preclinical studies using human xenografts in mice is, that the formation of new vessels in the tumors might be of both human and mouse origin. The interaction between mouse host stroma and human tumor cells and the role they play in the tumor-angiogenesis and tumor growth are of great importance in antiangiogenesis treatment. Therefore in preclinical studies using antiangiogenic drugs for treatment, it is of great importance to take into consideration species specificity. Accordingly, the antibody Bevacizumab only binds to human VEGF-A and not to murine VEGF-A [31], whereas the peptide Cilengitide is blocking Integrin *α*_V_ *β*_3_ of both human and mouse origin [32]. In the present study we therefore quantified both the gene expression level of mice and human Integrin *α*_V_, Integrin *β*_3_, and VEGF-A in our human H727 xenograft model. We found that Integrin *α*_V_ and Integrin *β*_3_, of both mouse and human origin, are present at comparable levels. In contrast, VEGF-A is mainly of human origin. In general this seems to be true throughout tumor development as we previously (unpublished data) found dual origin from 1–3 weeks of tumor growth with a confirmed higher level of VEGF-A of human origin. Species specificity of our PCR designs were verified targeting human or mice gene sequences during blast tests, and as a further control QPCR primers targeting mice genes were tested in cDNA from a NET patient finding no gene expression. 

The finding of correlation between tracer uptake of ^64^Cu-NODAGA-c(RGDyK) and mRNA levels of mouse Integrin *α*_V_, Integrin *β*_3_, and VEGF-A two hours post injection indicates that ^64^Cu-NODAGA-c(RGDyK) is indeed targeting the tumor-angiogenesis and therefore is a promising radiotracer for PET imaging and quantification of angiogenesis. 

Taken together ^64^Cu-NODAGA-c(RGDyK) seems promising for future use in humans. We therefore performed experiments to estimate dosimetry in humans based on projections from animal data. Here we found a favorable estimate of less than 8 mSv for a realistic clinical dose of 200 MBq. It should be noted that this estimate is conservative. Compared to recent dosimetry estimates for ^68^Ga-NODAGA-c(RGDyK) [9] on a per MBq basis the radiation burden is comparable especially taking into consideration the more conservative urinary elimination model used in our calculations. In both studies the organ receiving the highest radiation-absorbed dose was the urinary bladder wall.

## 5. Conclusion

We, as the first, have described the use of ^64^Cu-NODAGA-c(RGDyK) as a PET tracer targeting angiogenesis. We found an excellent tumor-to-background ratio due to rapid elimination from nontumor tissue. Also an excellent image quality was obtained using ^64^Cu. In addition, our compound seems stable as indicated by the low liver uptake. Using gene expression analyses we found a close correlation with key angiogenesis markers validating the target of the tracer. Finally the tracer had a favorable dosimetry. We therefore suggest that ^64^Cu-NODAGA-c(RGDyK) should be further studied as a promising candidate for human angiogenesis PET.

## Figures and Tables

**Table 1:** Optimized primer- and TaqMan probe concentrations for all genes investigated using quantitative real-time PCR.

Gene	NM no	Forward primer	Reverse primer	TaqMan probe
		Final conc., nmol/L	Final conc., nmol/L	Final conc., nmol/L
h-ITGAV	NM_002210	300	600	300
h-ITGB3	NM_000212	300	300	300
h-VEGFA	NM_001025366	300	600	300
m-ITGAV	NM_008402	600	600	300
m-ITGB3	NM_016780	300	300	300
m-VEGFA	NM_001025250	300	300	250
h-HPRT	NM_000194	300	300	200
h-UBC	NM_021009	300	300	200
h-RPLP	NM_001002	300	300	150

**Table 2:** Final QPCR designs for human and mice genes of interest and for housekeeping genes.

Gene	NM no	Forward primer, 5′-3′	Reverse primer, 5′-3′	5′-Flourophore	TaqMan probe, 5′-3′	3′-Quencher	Amplicon length (bp)
Humane							

h-ITGAV	NM_002210	GGGTCAAGATCAGTGAGAAATCTTTAC	ATTCCTGTAACATCATGCTATTGCTAG	FAM	AGGAACCTGGACCCCTTACCCCAACTTT	BHQ-1	139
h-ITGB3	NM_000212	CTCCTGTCCCTCATCCATAGC	CAGCCAAGAGGTAGAAGGTAAATAC	CY5	ACAGCACACCAAGGCACAGGGC	BHQ-2	103
h-VEGFA	NM_001025366	GTGTGAGTGGTTGACCTTCCTC	CCGTATATAAAACACTTTCTCTTTTCTCTG	HEX	CCTGGTCCTTCCCTTCCCTTCCCGA	BHQ-1	125

Mice							

m-ITGAV	NM_008402	ACGTTACATAGCATAGTACCTCTTC	TACTGATGGTCTAAATTTGAACTGC	FAM	TGCCCTAACGTAACGTCGAAGAGAGAAACC	BHQ-1	121
m-ITGB3	NM_016780	TCAACATCCTAAGATGCCGAGAG	CTGCTTGTTCTACTACTGGTCAAC	CY5	ACGGTATCAAGCCTGCGGTCCAGATTC	BHQ-2	104
m-VEGFA	NM_001025250	GGGACCCCTTCGTCCTCTC	GTCTCCTGGGGACAGAATTAGTG	HEX	ATCACACAAGGTCCTCCTGGGCTGT	BHQ-1	100

Housekeeping genes							

h-HPRT	NM_000194	AAGCCTAAGATGAGAGTTCAA	GCTCTACTAAGCAGATGGC	FAM	AACATCTGGAGTCCTATTGACATCGC	BHQ-1	108
h-UBC	NM_021009	CTGGAAGATGGTCGTACC	GTCAGGGTCTTCACGAAG	HEX	CACCTCTGAGACGGAGCACC	BHQ-1	107
h-RPLP	NM_001002	TGTGCAGCTGATCAAGAC	AGCACTTCAGGGTTGTAG	CY5	CTGCCATTGTCGAACACCTGC	BHQ-2	141

Table 2 shows the primers and probe sequences, the flourophores and quenchers used on the TaqMan probes and the amplicon length of the product for each gene.

**Table 3:** Residence times (Bq ∗ h/Bq) in the main target organs.

Target organ	Residence time [Bq ∗ h/Bq]
Liver	3.00*E* − 01
Kidneys	2.10*E* − 01
Lungs	1.29*E* − 02
Spleen	1.20*E* − 02
Heart	3.54*E* − 03
Small intestine	2.79*E* − 01
Remainder body	1.36*E* + 01

Data based on average biodistribution in organs at 1, 2, and 18 hours after injection obtained in human neuroendocrine xenograft tumor mice.

**Table 4:** OLINDA estimated radiation-absorbed doses for human adults after injection of  ^64^Cu-NODAGA-c(RGDyK).

Target organ	Organ doses (mGy/MBq)	
	Adult female	Adult male
Adrenals	1.45*E* − 04	1.14*E* − 04
Brain	1.25*E* − 04	9.76*E* − 05
Breasts	1.18*E* − 03	9.23*E* − 04
Gallbladder wall	0.00*E*000	0.00*E*000
Lower large intestine wall	3.93*E* − 03	3.10*E* − 03
Small intestine	2.88*E* − 04	2.46*E* − 04
Stomach wall	3.43*E* − 03	2.71*E* − 03
Upper large intestine wall	1.58*E* − 04	1.25*E* − 04
Heart wall	0.00*E*000	0.00*E*000
Kidneys	3.48*E* − 04	3.16*E* − 04
Liver	1.28*E* − 03	9.73*E* − 04
Lungs	1.10*E* − 03	8.68*E* − 04
Muscle	1.34*E* − 04	1.06*E* − 04
Ovaries/testicles	6.62*E* − 03	5.28*E* − 03
Pancreas	1.48*E* − 04	1.17*E* − 04
Red marrow	2.72*E* − 03	2.19*E* − 03
Osteogenic cells	5.74*E* − 04	4.28*E* − 04
Skin	2.32*E* − 04	1.83*E* − 04
Spleen	7.83*E* − 05	6.37*E* − 05
Thymus	1.33*E* − 04	1.03*E* − 04
Thyroid	1.28*E* − 03	1.06*E* − 03
Urinary bladder wall	1.37*E* − 02	1.03*E* − 02
Uterus	1.79*E* − 04	1.46*E* − 04
Total body	0.00*E*000	0.00*E*000
Effective dose (mSv/MBq)	3.78*E* − 02	2.94*E* − 02

Estimated doses based on average biodistribution in organs at 1, 2, and 18 hours post injection obtained in human neuroendocrine xenograft tumor mice.
